# High ^18^F-Fluorodeoxyglucose Uptake in Adrenal Angiomyolipoma: Case Report and Review of Literature

**DOI:** 10.1097/MD.0000000000000900

**Published:** 2015-06-05

**Authors:** Wenbo Li, Hua Pang, Youde Cao, Lili Guan, Jing Chen

**Affiliations:** From the Department of Nuclear Medicine (WL, HP, LG, JC); Institute of Pathology, The First Affiliated Hospital of Chong Qing Medical University, Chongqing 400016, China (YC).

## Abstract

Adrenal angiomyolipoma is an extremely rare tumor, although computed tomography (CT) or magnetic resonance imaging findings of adrenal angiomyolipoma have been reported, there are no reports regarding integrated fluorine-18-fluorodeoxyglucose positron emission tomography and computed tomography (^18^F-FDG PET/CT) imaging. We report a case of adrenal angiomyolipoma showing a significantly high uptake of ^18^F-fluorodeoxyglucose on PET/CT study. The maximal standardized uptake value (SUV) of the lesion was 18.8.

Adrenal angiomyolipoma can show an intense uptake in FDG-PET/CT, and this can easily be confused with a malignant disease. Adrenal angiomyolipoma should be considered as one of the differential diagnoses in cases of adrenal incidentaloma with intense FDG uptake.

## INTRODUCTION

Adrenal masses are seen in a wide variety of conditions, including benign lesions such as pheochromocytoma adrenal tuberculosis granulomatous infections and malignant diseases, and primary adrenocortical carcinoma and especially adrenal metastasis. Since adrenal metastasis may be encountered in a patient with either a known or an unknown primary malignancy, high uptake in an adrenal mass on fluorine-18-fluorodeoxyglucose positron emission tomography and computed tomography (18F-FDG PET/CT) is thought to represent malignancy. We present a case of adrenal angiomyolipoma that showed intense FDG uptake, and a review of the literature.

## CASE REPORT

Specifically, a 53-year-old male patient was admitted to our institution on December 03, 2013, with a 6 months history of abdominal pain in the upper left abdomen.

The patient's vital signs were stable at admission, and physical examination showed weakness and tenderness in the upper left abdominal quadrant.

**FIGURE 1:**
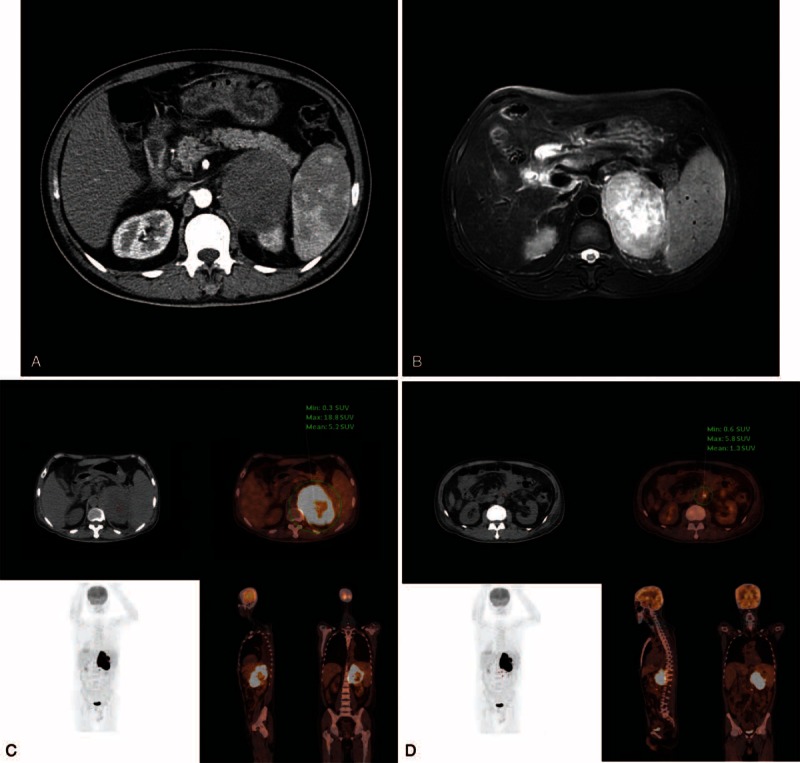
(A) Abdominal contrasted enhanced CT demonstrated a low-density mass in the left adrenal gland, with loss of fat planes between the mass and the kidney. (B) On magnetic resonance imaging, T2-fatsat images showed a soft tissue mass in the area of the left adrenal gland, edge smooth, mixed tumor of high signals, closely related to spleen, the intervening fat plane was effaced. (C). FDG PET/CT showed a significantly increased FDG uptake in the left adrenal gland with a maximal standardized uptake value (SUV) of 18.8. (D) A second small hypermetabolic presumed metastatic lesion, with SUV of 5.8. CT =  computed tomography, FDG = fluorine-18-fluorodeoxyglucose, PET/CT = positron emission tomography/computed tomography.

Abdominal ultrasonography revealed a well-defined 9 × 6 cm mass in the left adrenal gland. Baseline hematological, and biochemical investigations and urinalysis were normal. Laboratory investigations of serum cortisol, aldosterone, catecholamine, plasma adrenocorticotropic hormone, vanilmandelic acid, 24 hours urinary free cortisol, and a test for low-dose dexamethasone suppression were also within normal limits. On enhanced CT imaging, a low density mass was noted in the left adrenal gland with loss of fat plane between the mass and the left kidney (Figure 1A). On magnetic resonance imaging, T2-fatsat images showed a soft tissue mass in the area of the left adrenal gland, edge smooth, mixed tumor of high signals, closely related to spleen, the intervening fat plane was effaced (Figure 1B). Since clinical signs of Cushing syndrome were absent, adrenal incidentaloma primary adrenocortical carcinoma and metastatic adrenal lesions were suspected. Subsequently, whole-body 18F-FDG PET/CT was performed to characterize the adrenal lesion and to look for additional lesions. FDG PET/CT (Figure 1C and D) showed a significantly increased FDG uptake in the left adrenal mass suggestive of a malignancy. An additional hypermetabolic lesion was noted in the retroperitoneum which was thought to represent metastasis. The maximal standardized uptake value of the left adrenal mass was 18.8 and the metastatic lesion was 5.8.

**FIGURE 2:**
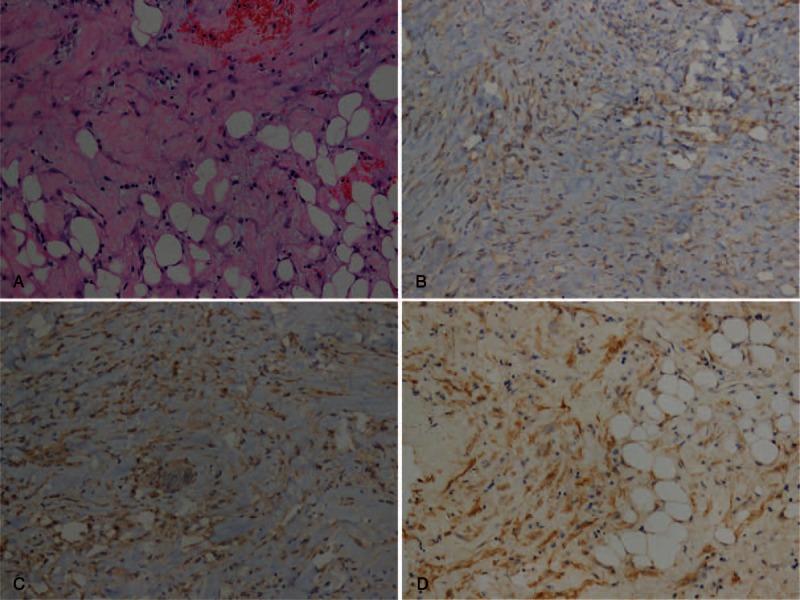
Hematoxylin and eosin staining (×200) showed that the tumors were composed of mature adipose fat cells, bundles of smooth muscle, thick-walled blood vessels with peripherally compressed adrenal cortical tissue suggestive of angiomyolipoma of adrenal (A). Tumor cells exhibited diffuse immunoreactivity for human melanoma black-45 (B), Melan-A (C), and smooth muscle actin (D) confirmed the diagnosis of adrenal angiomyolipoma.

Laparoscopic adrenalectomy with adrenal mass resection was performed after having provided informed consent with the patient. The histopathological features (Figure 2) confirmed the diagnosis of adrenal angiomyolipoma. The patient made an uneventful recovery and was normal at the 8-month follow-up period.

## DISCUSSION

Angiomyolipomas belong to the group of tumors that exhibit a diverse appearance known as tumors of perivascular epitheloid cell origin. Angiomyolipomas are rare lesions, and most occur in the kidney. The next common site is the liver. Adrenalangiomyolipoma is an extremely rare tumor, and to our knowledge only 10 cases have been reported, including the present case.^1–8^ In our specific reported case, the tumor size is 9 × 6 cm, and is the first reported case using combined 18F-FDG PET/CT imaging. The typical lesion of angiomyolipoma is composed of an admixture of thick-walled blood vessels, bundles of smooth muscle, and mature adipose tissues. Largely through the research of a group of pathologists, in recent years, it has become evident that the most distinctive component of this tumor is immunoreactivity for HMB-45.^9^ Up to 52% of patients with angiomyolipomas larger than 4 cm were symptomatic and had an increased risk of spontaneous rupture and bleeding. In addition, it has been suggested that in large angiomyolipomas, the risk of malignancy increases with the size of the tumor, following surgery or selective arterial embolization.^7–8^ In recent years, laparoscopic adrenalectomy has been recommended with lower mortality as compared with open surgery.

## CONCLUSIONS

In conclusion, adrenal angiomyolipomas can show an intense uptake in FDG-PET/CT, and can easily be confused with a malignant disease. Adrenal angiomyolipoma should be considered as one of the differential diagnoses in cases of adrenal incidentaloma with intense FDG uptake.
